# Exploring booster vaccine hesitancy: evidence from the COVID-19 in Hong Kong

**DOI:** 10.1080/16549716.2026.2750708

**Published:** 2026-10-22

**Authors:** Jiahui Jin, Daniel W.L. Lai, Elsie Yan

**Affiliations:** a Wee Kim Wee School of Communication and Information, Nanyang Technological University, Singapore, Singapore; b Faculty of Arts and Social Sciences, Hong Kong Baptist University, Hong Kong, SAR, China; c Department of Applied Social Sciences, The Hong Kong Polytechnic University, Hong Kong, SAR, China

**Keywords:** COVID-19, pandemic, vaccine, additional dose, daily hassles

## Abstract

**Background:**

The COVID-19 pandemic showed that effective pandemic prevention may require repeated vaccination. However, booster uptake can be influenced by psychosocial factors, particularly during a prolonged public health crisis.

**Objective:**

This study examined factors associated with adults’ willingness to receive an additional COVID-19 vaccine dose in Hong Kong.

**Methods:**

Data were drawn from the fourth-wave follow-up survey of a longitudinal study and analyzed cross-sectionally using multinomial logistic regression.

**Results:**

A total of 689 participants were analyzed: 401 (58.2%) were willing to receive an additional dose, 130 (18.9%) refused, and 158 (22.9%) were undecided. Relative to being undecided, higher pandemic fatigue (OR = 1.630, 95% CI: 1.256–2.115) and lower trust in authorities (OR = 0.602, 95% CI: 0.471–0.769) were statistically significantly associated with greater odds of refusal, whereas greater perceived convenience of preventive measures was statistically significantly associated with greater odds of acceptance (OR = 1.288, 95% CI: 1.011–1.640). Higher daily hassles were statistically significantly associated with increased odds of both refusal (OR = 1.578, 95% CI: 1.150–2.163) and acceptance (OR = 1.257, 95% CI: 1.005–1.572), indicating a bidirectional relationship, and the association with acceptance held only among those who perceived measures as less convenient. Among demographic factors, only age was significant, with younger participants more likely to remain undecided than older participants.

**Conclusions:**

The findings highlight the role of everyday pandemic experiences in shaping vaccination decisions. Improving the convenience and manageability of preventive measures may help promote booster vaccine uptake in future pandemic contexts.

## Paper Context

**Main findings**: Pandemic fatigue, lower trust in authorities, daily hassles, and perceived inconvenience of preventive measures were associated with reduced willingness to receive an additional coronavirus vaccine dose among adults in Hong Kong.

**Added knowledge**: This study shows that booster vaccination decisions during prolonged public health crises are shaped not only by risk perceptions, but also by people’s everyday experiences of fatigue, inconvenience, and trust.

**Global health impact for policy and action**: Future vaccination strategies should reduce the practical and psychological burden of prevention by making protective measures more convenient, manageable, and trustworthy.

## Background

Vaccination is a recognized method for reducing the spread of infectious epidemics (e.g. influenza, SARS, and COVID-19) [1–3], and many countries and jurisdictions are actively developing and promoting vaccine programs during epidemics for mitigating the adverse consequences. However, vaccine hesitancy emerged among populations, which the World Health Organization (WHO) considers a severe threat to public health, especially during large-scale epidemics [4]. Take COVID-19 as an example: for every 1% reduction in vaccine hesitancy, up to 45 deaths per million individuals could be avoided [5]. Over the past few decades, a number of scholars have summarized a range of determinants of vaccine hesitancy, including socio-cultural context, public trust, personal knowledge, accessibility of vaccines, etc. [6,7]. The global outbreak of COVID-19 that began in late 2019 brought the issue of vaccine hesitancy to the forefront of public attention again. However, in the context of COVID-19, vaccine hesitancy has become more complex due to concerns about adverse reactions, uncertainties regarding vaccine efficacy, and the need for multiple vaccinations within a short time frame [8–10]. Because the efficacy of the COVID-19 vaccine declines over time [11,12] and viruses continuously mutate [13], booster doses every 6 months are strongly recommended [14,15]. Since the worldwide implementation of the COVID-19 vaccine program began at the end of 2020, individuals were asked by local governments or health authorities to take ≥2 doses, while these repeated suggestions did not elicit a positive response [16]. Thus, investigating the factors related to the intention to take extra vaccine doses is instrumental in identifying barriers and developing vaccination promotion strategies. Large-scale pandemics like COVID-19 are unlikely to be a thing of the past. Some scholars predict that human beings will continue to experience occasional outbreaks of similarly infectious diseases in the future [17]. At that point, vaccination will remain the most effective strategy. Repeated use of vaccines, while clinically effective, may continue to face challenges and resistance [10,18]. These findings from COVID-19 have implications for vaccine implementation strategies during future pandemics, which are likely to recur [17].

Studies focusing on COVID-19 vaccination uptake have extensively examined factors like safety, effectiveness, affordability, and necessity [19–24]. Moreover, knowledge or perception is constructed based on personal experience, and incomplete experience can lead to distorted or inaccurate perceptions [25]. Therefore, the effects of personal experiences on self-protective behavior have been suggested, especially in the decision-making research field [26]. Experience-based factors, like daily life disruptions caused by the pandemic, have not been thoroughly studied in predicting vaccine intentions during the COVID-19 crisis. Hence, this study aims to incorporate experience-based factors into the self-determination theory framework to explore the elements linked with hesitancy toward ongoing vaccination.

Literature on vaccination uptake has revealed the relevance of the Self-Determination Theory (SDT) in explaining an individual’s motivation to make changes driven by the need for autonomy, competence, and relatedness [27]. Autonomy involves the need for individuals to feel free from coercion, whereas competence refers to the need to experience mastery [28]. Convenience, defined as the ease and accessibility of preventive measures, is related to autonomy, implying that it is a necessary component of an unrestricted environment for behavioral decision-making [29]. Moreover, convenience was found to be a moderator, which helps to strengthen the effect of psychosocial need satisfaction on decision-making [30].

Daily hassles were reported to be associated with both autonomy and competence [31], as they can stem from an individual’s personal abilities as well as from external constraints imposed by various measures or policies. Previous studies have also shown that daily hassles during the COVID-19 pandemic and the perceived level of convenience of pandemic prevention and control measures (e.g. mask wearing) were associated with antipandemic fatigue [32,33], while fatigue was found to be the primary reason that led to low vaccination intentions in view of desensitization toward risk after long-term exposure [5]. Earlier studies also indicated that mental fatigue appears when cost is high, but benefits are limited [34]. Such unsatisfactory outcomes would lead to less perceived competence, which would weaken the motivation for behavior uptake in the SDT framework [28].

Relatedness refers to feelings of closeness and belonging to a social group, which is a critical part for individuals to get social support, and trust is the bond that nurtures the sense of belonging and attachment to society [28]. The WHO Strategic Advisory Group of Experts on vaccine hesitancy stated the ‘3 Cs’ model, which highlighted that trust in the government or medical institution promoting the vaccine strongly affects an individual’s decision to be vaccinated [7]. Although public trust has been examined previously [35], indecision about accepting vaccination is highly dependent on context, such as social norms and peer influence [7]. Continued study of trust in authorities across different contexts, therefore, remains valuable.

Vaccine hesitancy is well documented in Hong Kong, where local studies reported that acceptance of COVID-19 vaccines declined notably as the pandemic progressed, falling to 37.2% [36], well below the recommended uptake of at least 55% [37], making it a useful setting for examining this issue. Drawing on relevant theory and prior findings on vaccine uptake, this study investigated the effects of daily hassles, pandemic fatigue, trust in authorities, and the perceived convenience of pandemic prevention and control measures on the intention to receive a booster vaccine dose.

Four hypotheses were proposed in this study: **H1)** a higher level of daily hassle decreases the odds of receiving an additional vaccine dose; **H2)** a higher level of pandemic fatigue decreases the odds of receiving an additional vaccine dose; **H3)** a higher level of trust in authorities increases the odds of receiving an additional vaccine dose; and **H4)** a higher perceived level of convenience of pandemic prevention and control measures increases the odds of receiving an additional vaccine dose.

## Methods

### Research design

This population-based longitudinal survey was conducted among Chinese adults residing in Hong Kong at 4 time points (T1–T4), corresponding to sequential waves of data collection: T1 (December 2020–January 2021) was the baseline survey, T2 (June 2021–July 2021) and T3 (December 2021–January 2022) were follow-up waves, and T4 was the fourth-wave follow-up survey. The present study analyzed data cross-sectionally from the T4 survey (between March 2022 and April 2022, *n* = 703), collected during the most severe period of the fifth wave of the COVID-19 pandemic in Hong Kong, with nearly 140,000 reported new infections between 22 March and 18 April 2022.

Telephone numbers were obtained through a multi-stage random-digit dialing procedure, in which valid number combinations were produced at random and dialed [38,39], rather than drawn from any existing list or private database, so no pre-existing private contact information was accessed. Individuals reached in this way were invited to participate and provided verbal informed consent. This population-based longitudinal survey was conducted among Chinese adults residing in Hong Kong, defined as ethnically Chinese residents aged 18 years or above. The term refers to ethnicity and residency rather than citizenship or nationality. Ethnic Chinese constitute the large majority of Hong Kong’s population, and the survey was administered in Cantonese; the study population was defined accordingly to reflect the general adult population and to allow consistent survey administration in a common language.

A total of 2784 telephone numbers were generated for this study, and 1255 eligible individuals completed the initial data collection (T1). In the subsequent data collection phases, the project had 1003 participants in T2 and 803 participants in T3. For the T4 data used, all 803 participants from T3 were contacted, and 703 participants completed the data collection (response rate = 87.5%).

This study was approved by the Human Subject Ethics Sub-Committee of the Hong Kong Polytechnic University (Approval Number: HSEARS 20200814002). This study was conducted in accordance with the principles of the Declaration of Helsinki. Verbal informed consent was obtained from all participants prior to data collection. This approach was approved by the Ethics Committee and was used because data collection was conducted by telephone, making written consent impractical. All data were de-identified prior to analysis, and only the de-identified dataset was used for analysis, so no author had access to information that could identify individual participants.

### Measurements

Dependent variables were measured by asking the participants about their willingness to receive an additional vaccine dose because of the fifth wave of the COVID-19 pandemic in Hong Kong. Available choices for the question included ‘yes,’ ‘no,’ and ‘undecided.’ The full wording of the outcome question and all measurement items is provided in the Appendix.

Daily hassles due to the pandemic were measured with 10 items covering diverse life domains (employment or study, social activities, leisure, economic circumstances, and going out). The items were developed for this study by the research team, drawing on daily difficulties commonly reported in local media, and were not adapted from a previously validated instrument. Each item was rated from 1 (never) to 5 (always), and a composite score was calculated as the mean of the 10 items (range 1–5), with higher scores indicating greater hassles. Internal consistency was high (Cronbach’s *α* = .901).

Pandemic fatigue was measured with eight items adapted from the 10-item Fatigue Assessment Scale [40], a validated instrument, capturing emotional responses to the pandemic (e.g. worry, low energy). Each item was rated from 1 (never) to 5 (always), and a composite score was computed as the item mean, with higher scores indicating greater fatigue (*α* = .957).

Trust in authority was measured with two items developed for this study, rating trust in the government and trust in medical experts to control COVID-19, each from 1 (absolutely distrust) to 10 (absolutely trust). A composite score was computed as the mean of the two items (*α* = .775).

Perceived convenience of the pandemic prevention and control measures was measured using a 12-item scale, with each item rated from 1 (very inconvenient) to 5 (very convenient). The 12 items were developed by the research team to reflect the convenience of the specific prevention and control measures in place during the study period (e.g. mask wearing, the vaccination pass, gathering restrictions), drafted on substantive grounds to match the local context. A composite score was computed as the mean of the items, with higher scores indicating greater perceived convenience. The scale showed satisfactory internal consistency (*α* = .941).

Additionally, questions on demographic variables, including age, sex, education, involvement in economic activity, and the number of vaccination doses taken at the time of the survey, were collected. All this information is collected during each data collection to ensure the most up-to-date demographic backgrounds. Economic activity was measured by the participant’s employment status (e.g. employed, a student, unemployed, or retired). Subsequently, ‘employed’ was categorized as active economic activity, while all the other responses were classified as inactive.

### Data analysis

Data analysis was performed using SPSS version 26.0. Descriptive statistics were used to calculate the distribution of demographic variables (i.e. gender, age group, economic activity status, and educational attainment), including the frequency and percentage distributions. Missing data were examined prior to analysis. Of the 703 respondents who completed the T4 survey, 14 (2.0%) had missing values on the outcome variable (willingness to receive an additional dose) and were removed via listwise deletion, yielding an analytic sample of 689. The outcome variable was willingness to receive an additional vaccine dose, a categorical variable with three unordered categories (yes, no, and undecided). Because this outcome was categorical rather than continuous, multinomial logistic regression, rather than linear regression, was used, with ‘undecided’ as the reference category. The predictors were the four independent variables (daily hassles, pandemic fatigue, trust in authority, and perceived convenience), with demographic variables (age, sex, educational attainment, economic activity, and number of doses received) included as covariates. In a logit model, odds ratios (OR) and 95% confidence intervals (CI) with *p*-values were presented. A *p*-value of <0.05 was used to indicate significance. The goodness of fit of the multinomial logistic model was assessed by the likelihood-ratio (model-fitting) chi-square test, which compares the fitted model with the intercept-only null model, and model goodness-of-fit was assessed using the Pearson chi-square goodness-of-fit test. Demographic variables were included in a multinomial logistic model as confounding variables. For results showing an unexpected direction in the relationship between the independent and dependent variables, moderating effects of the level of perceived convenience were further examined using two subsets of participants, as suggested by previous research [30]. To probe this, we split the sample into two subsets, each excluding one outcome group so that a single pairwise contrast remained: one subset excluded participants who refused the dose (leaving acceptance versus undecided), and the other excluded those willing to receive it (leaving refusal versus undecided). Isolating each contrast in this way allowed the interaction between the predictor and perceived convenience to be tested within a binary logistic regression, giving a clearer picture of how the predictor related to each attitude. Where a significant interaction emerged, we probed it by estimating the conditional (simple-slope) effect of the predictor at low, medium, and high levels of the moderator (the 16th, 50th, and 84th percentiles).

## Results

### Participants and extra vaccination dose

Table 1 illustrates the demographic characteristics of the analytic sample (*n* = 689). The last column of Table 1 displays the gender and age distribution of the Hong Kong population in 2020 [41]. The research sample’s results are comparable to the regional census data. Among the participants, 401 (58.2%) indicated their willingness to receive an additional vaccine dose, 130 (18.9%) refused, and 158 (22.9%) were undecided. At the time of data collection, all the participants had received at least one dose of the vaccine, owing to the compulsory ‘Vaccine Pass’ policy implemented by the government in February 2022.

**Table 1.** Participant characteristics and study variables (analytic sample, *n* = 689).

		Count (%)	Gender and Age in Hong Kong population*
Gender	Male	337 (48.9%)	47.0%
	Female	352 (51.1%)	53.0%
Age	Aged 18–34	162 (23.5%)	23.7%
	Aged 35–54	239 (34.7%)	35.0%
	Aged 55 and above	288 (41.8%)	41.3%
Economic activity status	Active	464 (67.3%)	
	Inactive	225 (32.7%)	
Educational attainment	Lower secondary or below	221 (32.1%)	
	Upper secondary	307 (44.6%)	
	Diploma/degree or above	161 (23.4%)	
Willingness to receive an additional dose		Count (%)	Mean (SD)
Accept		401 (58.2%)	
Undecide		158 (22.9%)	
Refuse		130 (18.9%)	
Daily hassles			3.42 (0.874)
Pandemic fatigue			2.35 (0.791)
Trust in authority			3.24 (1.089)
Perceived convenience			3.33 (0.888)

Note: SD = standard deviation. *Based on data estimates from Women and Men in Hong Kong Key Statistics 2020 Edition.

### Results of multinomial logistic regression

Table 2 presents the result of the multinomial logistic regression. The likelihood-ratio model chi-square indicated that the fitted model provided a significantly better fit than the null model (*x*^2^ = 102.266, df = 24, *p* < 0.001). The Pearson chi-square goodness-of-fit test was non-significant (*x*^2^ = 1346.296, df = 1346, *p* = 0.493), indicating adequate model fit. Among the demographic variables entered in the model, age was found to have a significant impact on the participants’ willingness to receive an extra vaccine dose. Younger participants were more likely to be undecided.

**Table 2.** Multinomial logistic regression of factors associated with willingness to receive an additional COVID-19 vaccine dose (reference group: undecided).

	No		Yes	
	*B* (SE)	OR (95% CI)	*B* (SE)	OR (95% CI)
Hassles	.456** (.161)	1.578 (1.150–2.163)	.229* (.114)	1.257 (1.005–1.572)
Pandemic fatigue	.488*** (.133)	1.630 (1.256–2.115)	−.174 (.106)	.840 (.683–1.033)
Trust in authority	−.508*** (.125)	.602 (.471–.769)	−.151 (.101)	.860 (.705–1.049)
Measures’ convenience level	−.084 (.152)	.919 (.682–1.238)	.253* (.123)	1.288 (1.011–1.640)
Gender				
Male^1^	−.127 (.258)	.881 (.532–1.459)	.366 (.203)	1.442 (.969–2.145)
Age				
Aged 18–34^2^	−1.274** (.410)	.280 (.125–.624)	−.711* (.319)	.491 (.263–.917)
Aged 35–54^2^	−.959** (.348)	.383 (.194–.758)	−.578* (.266)	.561 (.333–.945)
Educational attainment				
Lower secondary or below^3^	−.719 (.464)	.487 (.196–1.209)	.194 (.360)	1.214 (.600–2.458)
Upper secondary^3^	−.600 (.337)	.549 (.284–1.062)	−.379 (.272)	.684 (.402–1.166)
Economic activity status				
Active economic activity status^4^	.243 (.310)	1.275 (.694–2.339)	.061 (.243)	1.063 (.660–1.710)
Vaccinated 1 dose^5^	−.965 (.702)	.381 (.096–1.507)	.365 (.489)	1.440 (.552–3.757)
Vaccinated 2 doses^5^	−.135 (.266)	.874 (.518–1.472)	.440* (.211)	1.553 (1.027–2.349)
Cox and Snell *R*^2^		0.138		
Nagelkerke *R*^2^		0.161		

Notes: *B* = unstandardized regression coefficient; SE = standard error; OR = odds ratio; CI = confidence interval. The outcome was willingness to receive an additional COVID-19 vaccine dose, coded as yes (willing to receive; *n* = 401), no (unwilling/refused; *n* = 130), and undecided (*n* = 158), with Undecided as the reference category. ^1^ref: female, ^2^ref: aged 55 and above, ^3^ref: diploma/degree or above, ^4^ref: inactive economic activities status, ^5^ref: vaccinated three doses. *p < .05, **p < .01, ***p < .001.

Compared with those who were undecided, participants who were unwilling to take an extra vaccine dose reported a higher level of daily hassles (OR = 1.578, 95% CI: 1.150–2.163) and pandemic fatigue (OR = 1.630, 95% CI: 1.256–2.115). Higher pandemic fatigue was thus associated with greater odds of refusal relative to indecision, consistent with H2. Higher daily hassles were associated with greater odds of both refusal and acceptance relative to indecision. Thus, H1 was not supported. For every one-unit increase in trust toward the authority, individuals showed a 39.8% decrease in the likelihood (OR = 0.602, 95% CI: 0.471–0.769) of being unwilling to receive an additional vaccine dose, as opposed to maintaining an undecided stance. Higher trust in authorities was thus associated with lower odds of refusal relative to indecision, consistent with H3.

Compared with those who were undecided, participants who were willing to receive an extra vaccine dose reported higher levels of daily hassles (OR = 1.257, 95% CI: 1.005–1.572) and perceived level of convenience of the pandemic prevention and control measures (OR = 1.288, 95% CI: 1.011–1.640). Higher perceived convenience was thus associated with greater odds of acceptance relative to indecision, supporting H4. Meanwhile, compared to individuals who had taken three doses of vaccine, the odds of getting another dose were higher for those who had already had two shots (OR = 1.553, 95% CI: 1.027–2.349). A summary of the hypothesis tests is presented in Table 3.

**Table 3.** Summary of hypothesis testing (multinomial logistic regression; reference category: undecided).

Hypothesis	No vs. undecided, OR (95% CI)	Yes vs. undecided, OR (95% CI)	Result
H1	1.578 (1.150–2.163)	1.257 (1.005–1.572)	Not supported (bidirectional; both contrasts significant and positive)
H2	1.630 (1.256–2.115)	ns	Supported (via refusal contrast)
H3	.602 (.471–.769)	ns	Supported (via refusal contrast)
H4	ns	1.288 (1.011–1.640)	Supported (via acceptance contrast)

Note: ns denotes nonsignificant results.

### Results of moderation effect

The results of the multinomial logistic model indicate that a higher level of daily hassles is associated with both accepting and rejecting attitudes toward the extra vaccine dose. These findings are unexpected, as they suggest that a higher level of daily hassles can both increase and decrease the intention to take an extra shot. Thus, the potential moderating effects of other predictors on the relationship between daily hassles and the decision on extra vaccine dose were examined with two subsets of participants. The moderating effect of the perceived convenience level of the pandemic prevention and control measures on the relationship between daily hassles and the decision to receive one more vaccine dose was found in the subset that excluded participants who refused one more booster dose (*n* = 559, *x*^2^ = 12.401, *p* < 0.001). Higher levels of daily hassles increased the odds of accepting an extra vaccine dose (*B* = −.498, SE = 0.150, *p* < 0.001; accepting the extra dose as the reference) only in individuals who perceived a lower level of convenience of pandemic prevention and control measures, whereas for those who reported a higher level of convenience, such effects disappeared. Slope-test results indicated that only when individuals perceived a lower level of daily hassles would a higher level of perceived convenience of the pandemic prevention and control measures increase the odds of taking one more dose (*B* = −.688, SE = 0.207, *p* < 0.001; accepting the extra vaccine dose as the reference). However, no significant moderating effects were found in the subset that excluded participants who received one more vaccine dose.

## Discussion

Although COVID-19 around the world appears to be under control, future pandemics of similar global scale are likely to happen again [17]. The use of annual or multiple vaccinations is an inevitable strategy in view of other viral threats to humankind [42,43]. The findings of this study not only expand the existing body of research on COVID-19 vaccine hesitancy by elucidating the factors associated with vaccine acceptance and resistance but also offer critical insights for the promotion and implementation of vaccine programs that require repeat injections. The results of this research, obtained in the context of the COVID-19 pandemic, indicated that higher levels of antipandemic fatigue and less trust in authorities increased the odds of rejecting an additional vaccine, whereas higher daily hassles were associated with both acceptance and refusal relative to indecision. Regarding between-group differences, younger participants were more hesitant about receiving an extra vaccine dose than older participants, while those who had been vaccinated twice were more likely to accept one.

Based on the results of the literature search, there are limited studies on the predictors of willingness to sustain vaccination, which may be because booster doses have only been introduced for a short period. Although small studies have been conducted in the non-Western world, a high uptake level of the booster dose is critical for ending the COVID-19 pandemic [16]. The significance of this study is that it provides additional insights for policymakers seeking to promote booster vaccination rates in future pandemics.

Unlike previous findings on demographic variables [5,22], only age had a significant effect on the willingness to receive one more vaccine dose, whereas sex, educational attainment, and economic status were found to have no effect. Although the effect of age on vaccination hesitation is inconsistent, a younger age is generally associated with less willingness to be vaccinated [44]. There is also a greater tendency for younger age groups to remain undecided rather than refuse vaccination, as shown in this study. This could probably be related to vaccine promotion measures during the data collection phase, which coincided with the introduction of the ‘Vaccine Pass,’ a proof of COVID-19 vaccination aimed at controlling the spread of the virus in Hong Kong. Access to certain services, events, or locations was restricted to individuals possessing a ‘Vaccine Pass,’ while young people generally have a greater need and interest in accessing these venues than older cohorts. Thus, the paradox of refusing vaccination and requiring proof for daily social activities may have emerged, leading to a higher level of hesitation regarding vaccination. Individuals who had already received two doses of the vaccine were more willing to receive an additional dose, probably because a three-dose regimen would be considered the norm sooner or later. This is notable given the international consensus that individuals who received two doses of the COVID-19 vaccine were regarded as fully vaccinated, whereas those who had received only one dose were no more willing to receive an additional dose. Logically, trust in the vaccine is higher among individuals who have closely followed the practice of receiving three doses, which explains why this group preferred to receive an extra vaccine dose. Additionally, more than 1 year after the vaccination program was implemented, those who had still received only one dose can be speculated to hold a resistant attitude toward the vaccine.

A higher level of fatigue hinders willingness to be vaccinated. This finding supports the argument that prolonged time and effort can exacerbate fatigue [34]. After years of stringent prevention and control measures, the spread of the virus did not seem to have been effectively controlled; thus, the high level of fatigue is understandable. The disappointment caused by the perception of a never-ending pandemic, together with the hassles of prevention and control measures, probably explains the significant reduction in the public’s sense of competence in getting vaccinated to protect themselves, which is a main motivation for achieving self-determination [27,28].

Contrary to H1, daily hassles did not uniformly reduce willingness. Higher hassles raised the odds of both accepting and refusing relative to indecision, suggesting that everyday pandemic stress pushed individuals away from an undecided stance in both directions rather than uniformly discouraging uptake. This divergence was clarified by an interaction between daily hassles and perceived convenience: the odds of receiving an additional vaccine increased only when either daily hassles or perceived convenience was low. Because the ‘Vaccine Pass’ introduced during data collection restricted access to many venues, the hassles it created may have driven greater willingness among those who go out or dine out frequently. Conversely, when such hassles were limited, higher perceived convenience motivated vaccination, reflecting the greater autonomy described in the SDT model [29].

These findings on trust in the authority are similar to those of previous studies [7,35]. In this study, the high level of trust in the authorities weakened citizens’ resistance to government-recommended measures but failed to strongly predict the adoption of such measures. Although trust in the authorities increases compliance with the government’s recommendations regarding vaccination, dissatisfaction with the performance of current measures to cope with COVID-19 may decrease vaccination adherence [45,46]. Consistent with this, transparent post-vaccination surveillance, clear risk communication, and effective management of misinformation have been identified as important means of building public trust and improving vaccine acceptance [47]. Thus, the weak effect of trust in authority could be due to the poor performance of the never-ending restrictive pandemic prevention and control measures that have resulted in further hassles.

Despite its contributions, this study has several limitations. First, its cross-sectional design precludes causal inference, so the associations reported should be interpreted with caution. Second, the baseline response rate was 45.1%, below 50%, which raises the possibility of non-response bias; because the survey concerned a sensitive and attitudinally charged topic, willingness to participate may itself have been associated with vaccine attitudes, so the observed distribution of willingness and the estimated associations may not fully represent the target population, and the direction of any such bias cannot be determined. The small amount of missing data may likewise have affected the results, and future studies would benefit from strategies to improve participation and to compare respondents with nonrespondents. Third, although the demographic distribution of the sample was comparable to that of the population, the sample size may still yield somewhat unstable estimates. Fourth, some scales employed (e.g. daily hassle) were developed by the research team from stressors reported in local media rather than adapted from a validated instrument; although its internal consistency was high (*α* = .901), no factor analysis was conducted to confirm construct validity, so findings involving this measure should be interpreted with caution and the scale validated in future research. Fifth, several potentially relevant factors were not measured, including experience of infection, comorbidities, vaccine type, and previously experienced side effects, all of which may shape perceived hassles and willingness to receive further doses. Finally, the study was conducted in Hong Kong, which may limit the generalizability of the findings to other regions.

## Conclusion

This study examined individuals’ willingness to receive an additional vaccine dose, considered key to long-term protection against COVID-19 and potentially future pandemics. The study incorporated several factors that have rarely been examined in research on vaccination hesitancy. Age was the only significant demographic predictor, with younger participants more likely than older ones to remain undecided. Daily hassles showed a bidirectional association with willingness that was moderated by perceived convenience. Efforts to promote booster uptake should therefore reduce the daily burden of pandemic measures and improve their perceived convenience. These conclusions should be read in light of the study’s limitations, including its cross-sectional design, the use of a nonvalidated scale, and a single-region sample that may limit generalizability.

## Supplementary Material

Appendix.docx

## Data Availability

Data are provided upon reasonable request.

## Abbreviations

COVID-19: Coronavirus Disease 2019
SARS: Severe acute respiratory syndrome
WHO: World Health Organization
SDT: Self-Determination Theory
SPSS: Statistical Package for the Social Sciences
