# Supplementary material for: Exploring booster vaccine hesitancy: evidence from the COVID-19 in Hong Kong
Source: Glob Health Action. 2026 Oct 22;19(1):2750708. doi: 10.1080/16549716.2026.2750708 (PMC13700476; doi:10.1080/16549716.2026.2750708)
Supplement: Supplementary file 1 [file ZGHA_A_2750708_SM6174.docx]

**Details of Questionnaire Used**

**Daily Hassles**

Whether the following yours have been disturbed by COVID-19:

**(1 = never, 2 = sometimes, 3=regularly, 4=often, 5=always)**

1. Conducting leisure activities

2. Access to medical, social and other public services

3. Economic environment

4. Employment/working environment

5. Social events

6. Interaction with family members

7. Dining out

8. Local trip

9. Trip overseas

10. Use of IT (e.g. booking public services, shopping, communicating with others, etc.)

**Pandemic fatigue**

In the past month, have you felt any of the following in response to COVID-19?

**(1 = never, 2 = sometimes, 3=regularly, 4=often, 5=always)**

1. I am bothered by fatigue

2. I get tired very quickly

3. I don’t do much during the day

4. Physically, I feel exhausted

5. I have problems to start things

6. I have problems to think clearly

7. I feel no desires to do anything

8. Mentally, I feel exhausted

**Trust in authority**

**1 = absolutely distrust … 10 = absolutely trust**

1. How much trust do you have in the Hong Kong government officials, such as the Chief Executive and the Secretaries of Departments, who are responsible for handling the COVID-19?

2. How much trust do you have in the medical and scientific professionals who deal with COVID-19?

**Perceived convenience of the pandemic prevention and control measures**

How convenient the following statutory disease prevention measures are to the public?

1 = very inconvenient….. 5 = very convenient

1. Restrictions on partying

2. Compulsory wearing of masks

3. Restrictions on dining and listed premises (e.g. mandatory use of LeaveHomeSafe or registration of personal data, etc.)

4. Grounding orders or relocation to a quarantine center for specific persons

5. 14-day or 21-day mandatory quarantine of immigrants

6. Compulsory testing of specific persons

7. Persons working or attending classes in certain organizations or establishments are required to be vaccinated or tested

8. Introduction of vaccine passes

9. Mandatory closure of areas for testing

10. Compulsory grounding of all persons for testing

11. Compulsory vaccination for all (except those who are medically unfit)

12. Mandatory vaccination for specified persons (except those who are medically unfit)
